# Nutritional, functional, and sensorial properties of oat milk produced by single and combined acid, alkaline, α‐amylase, and sprouting treatments

**DOI:** 10.1002/fsn3.3171

**Published:** 2022-12-08

**Authors:** Nima Babolanimogadam, Hassan Gandomi, Afshin Akhondzadeh Basti, Mohammad J. Taherzadeh

**Affiliations:** ^1^ Department of Food Hygiene, Faculty of Veterinary Medicine University of Tehran Tehran Iran; ^2^ Swedish Centre for Resource Recovery University of Borås Borås Sweden

**Keywords:** acid treatments, alkali treatments, functional food, oat milk, sprouting treatments, α‐amylase treatments

## Abstract

In this study, the effects of different treatments of the oat slurry on the nutritional, functional, and sensorial properties of oat milk were evaluated. The sprouting and sprouting–acidic treatments have the highest oat milk yield (91.70%) and protein extraction yield (82.74%), respectively. The protein concentrations of alkali, sprouting–acidic, and α‐amylase–alkali treatments were significantly (*p* < .05) higher than other treatments. The alkali treatments showed higher fat content (0.66%). In addition, acidic and alkali treatments in single or combined with other treatments showed the highest dry matter and energy value. The carbohydrate content of α‐amylase–alkali treatment (4.35%) was higher than other treatments and also, all acidic treatments showed higher ash content (>1) compared to the other treatments. Furthermore, the sprouting–α‐amylase and acidic–α‐amylase showed the lowest starch (0.28%) and the highest reducing sugar content (3.15%) compared to the other treatments, respectively. Moreover, the α‐amylase–alkali treatment showed the highest total phenolic content and antioxidant activity (342.67 mg GAE/L and 183.08 mg BHT eq/L, respectively). Furthermore, sensory evaluation of most treatments showed acceptable scores (≥7) for consumers, especially in the case of α‐amylase, sprouting, and α‐amylase–sprouting treatments. Results show that the different treatments had different effects on the nutritional, functional, and sensorial properties of oat milk. In conclusion, from the nutritional and functional point of view, the two‐stage treatments were more effective than singular treatments on investigated factors proposing their application in functional plant milk preparation.

## Graphical abstract

The effects of phosphoric acid, sodium hydroxide, a‐amylase, and sprouting, and the combination of those treatments in the production of oat milk were studied, and two‐stage treatments showed more effectiveness on evaluated factors.

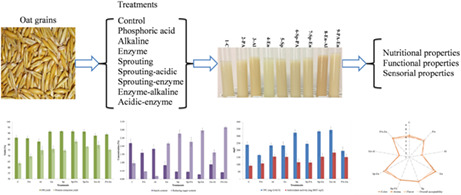

## 1 INTRODUCTION

Plant‐based milk alternatives are produced using the breakdown and extraction of plant material by various treatments (Sethi et al., 2016). This milk potentially contains nutritional and functional compounds according to the plant source and processing methods (Kumar et al., 2020; Mridula & Sharma, 2015). Nowadays, global consumption of plant milk is growing not only due to the environmental challenges of animal products but also due to the several functional properties and the more effective price of plant milk (Sethi et al., 2016).

Oat (*Avena sativa* L.) as a cereal grain has received increased interest due to its excellent nutritional profile, including protein with a balanced amino acid profile, unsaturated fatty acids, and dietary fiber (particularly, β‐glucan). It also contains bioactive phytochemicals, vitamins, phenolic compounds like phenolic acid and avenanthramides compounds, and other micronutrients (Grundy et al., 2018; Martínez‐Villaluenga & Peñas, 2017), which possess various biological and health beneficial effects, including antioxidant, anticancerous, antiallergic, anti‐inflammatory, and vasodilator effects, as well as prevention of type II diabetes and reduction in total serum cholesterol levels (Ding et al., 2019; Zhu et al., 2020). Oat milk (OM) is a water extraction of oat and is considered an emerging nutritional and bioactive food (Bocchi et al., 2021).

The composition of OM depends on the variety of oat grains used and the processing factors (Aparicio‐García et al., 2021; Xu et al., 2009; Yue et al., 2021). These factors affect not only the yield, sensorial, and rheological properties but also the nutritional profile of OM. For example, the yield, rheological, and sensorial properties of OM were affected by the gelatinization of the starch as the major portion of the oat during the thermal treatment (Punia et al., 2020; Zhu, 2017). In addition, phenolic components were found as bound or conjugated with structural fibers and proteins and remained in the pulp during extraction (Bei et al., 2017). Furthermore, proteins are mostly insoluble at slightly acidic or neutral pH (Brückner‐Gühmann et al., 2019), which leads to decreasing protein extraction yield. Thus, the high‐yield extraction of oat components as a challenge could be conducted with different treatments. Investigations showed that α‐amylase was used to partially remove the starch, which improved OM physicochemical properties (Deswal et al., 2014; Jeong et al., 2022). The treatments of sprouting, diluted acid, or alkaline were also found to be useful for extracting phenolic compounds through hydrolyzation and disrupting the cell wall of lignocellulosic materials (Chiranjeevi et al., 2018; Nascimento et al., 2016). Furthermore, mild alkaline treatments generally were used to isolate the proteins from the oat grains (Yue et al., 2021).

The aim of this study was to investigate on process optimization for achieving the highest nutritional yield and sensorial properties of OM by different treatments using hot water, acid, alkaline, α‐amylase, and sprouting, alone or in combination.

## 2 MATERIALS AND METHODS

### 2.1 Materials

The whole‐oat grains (WOG) were supplied from a local producer in Ardabil province, Iran. The α‐amylase was obtained from DSM Nutritionals (11,500 fungal amylase units (FAU)/g, Heerlen, Netherlands). All other chemicals were purchased from Merck (Darmstadt, Germany), if not otherwise stated.

### 2.2 Preparation of the treatments

After cleaning and separating of nonoat seeds, aliquots of 100 g of WOG were individually soaked in sodium hypochlorite (0.1%) at a ratio of 1:6 (w/v) for 30 min, and rinsed with sterile tap water four times for 5 min. Also, for sprouting, the grains were soaked in sterile tap water, at a ratio of 1:6 (w/v), for 48 h at room temperature, and drained grains were sprouted at 18°C for 96 h in darkness under a moist filter (Aparicio‐García et al., 2021). The sprouted and nonsprouted grains were dried at 50°C for 24 h, milled to obtain a particle size smaller than 0.5 mm, and stored in sterile bags at −18°C until use. Investigated treatments included control (C), acid (PA), alkaline (Al), enzyme (En), sprouting (Sp), and different combinations of treatments, including sprouting–acidic (Sp‐PA), sprouting–enzyme (Sp‐En), enzyme–alkaline (En‐Al), and acidic–enzyme (PA‐En). For all treatments, 500 ml of distilled water was added to 100 g milled oat in 1‐L bottles. The control treatment (C) was prepared by heating the slurry at 90°C for 10 min. The acid treatment (PA) was prepared by adding 5 ml of phosphoric acid (85%) and heating 80°C for 60 min, and the alkaline treatment (Al) was treated by adding 5 ml of NaOH (40% w/v) into the slurry. In addition, 200 mg of α‐amylase was employed for enzyme treatment (En) and heating for 60 min at 65°C. Sprouting treatments (Sp) were heated for 10 min at 90°C. Furthermore, the combination of these treatments was used for preparing sprouting–acidic (Sp‐PA), sprouting–enzyme (Sp‐En), enzyme–alkaline (En‐Al), and acidic–enzyme (PA‐En) treatments. In the case of all enzymatic treatments, the pH of the slurries was adjusted to 6.7 before the enzyme addition. After treatments, the weight of the slurries was adjusted to 1000 g and filtered manually with cheesecloth. Phosphoric acid (85%) and sodium hydroxide (40%) were used to adjust the pH to 6.7, and OM was heated for 10 min at 90°C. The OM produced by nine treatments was cooled and stored at 4°C until further analyses.

### 2.3 Proximate composition analysis

A pH meter (PB‐11, Sartorius, Germany) was used to evaluate pH at 25°C while stirring the OM samples. The Kjeldahl method was used for determining the protein content of milled WOG or OM samples (around 1 g or 5 ml, respectively) by using 6.25 as the conversion factor of the nitrogen to protein (Tian et al., 2010). The dry matter (DM), fat, and ash content of samples were determined according to the procedure described in AOAC (2000). Briefly, the DM content of samples was analyzed by drying preweighed samples (around 1 g or 10 ml for WOG or OM samples, respectively) at 105°C for 24 h. The fat content of samples was determined by Soxhlet systems with petroleum ether (BP 40–60°C) for 4 h extraction from dried samples. Furthermore, the ash content of samples was determined by the dry ashing method in preweighed porcelain crucibles by using 0.1 g of WOG or 1 ml of OM at 600°C for 4 h. The carbohydrate content was calculated by difference. Furthermore, the energy value (kcal per 100 g or 100 ml) of samples was calculated by using Equation (1) (WHO/FAO, 2003):

(1) $$\text{Energy value}=\left(\text{Protein}\%\times 4\right)+\left(\text{Carbohydrates}\%\times 4\right)+\left(\mathrm{Fat}\%\times 9\right)$$

### 2.4 Calculation of OM and protein extraction yield

The OM and protein extraction yield (PEY) were calculated by Equations (2) and (3), respectively (Yue et al., 2021):

(2) $$\mathrm{OM}\;\text{yield}\;\left(\%\right)=\left(W1/\text{W2}\right)\times 100$$

(3) $$\mathrm{PEY}\;\left(\%\right)=\left(\text{W1}\times \text{P1}\right)/\left(\text{W2}\times \text{P2}\right)\times 100$$

where W1 and W2 were the weight of extracted OM and the weight of oat grain (100 g), and P1 and P2 were protein concentrations of OM and oat grain, respectively.

### 2.5 Starch and reducing sugar determination

For determination of starch and reducing sugar content of the samples, milled WOG and OM samples were diluted with distilled water in a ratio of 1:50 (w/v) and homogenized by Homogenizer (Heidolph Instruments GmbH and CoKG, Germany) at 12,000 rpm for 1 min. The starch content of milled WOG and OM samples was determined according to Simsek et al. (2014) with some modifications. The 500 μl of iodine solution was mixed with the same volume of diluted samples. The optical density (OD) of the solution was measured at 620 nm using a microplate reader (BioTek Epoch 2 Microplate Spectrophotometer, Winooski, VT, USA). A standard curve was prepared using different concentrations of potato starch (0.0025%–0.05%), and the results were expressed as a percentage of the sample weight. Also, the dinitrosalicylic acid (DNS) method described by Miller (1959) was used for the evaluation of the reducing sugar content (RS) of WOG and OM samples with some modifications. Firstly, an aliquot of 1.5 g of DNS was added to 30 ml of the NaOH solution (2 M), and 45 g of sodium potassium tartrate was mixed separately in 75 ml of distilled water. Finally, the volume of the mixed solution was adjusted to 150 ml with distilled water. One ml of diluted samples was mixed with 1 ml of freshly prepared DNS reagent, incubated in a water bath for 15 min at boiling temperature, cooled to ambient temperature, and finally, adjusted to 10 ml using distilled water. The OD of the samples was measured at 540 nm. The calibration curve was obtained using glucose (0.0025%–0.1%), and the results were expressed as a percent.

### 2.6 Extraction of bioactive compounds

The method presented by Aparicio‐García et al. (2021) was used for the extraction of bioactive compounds of OM, with slight modification. The milled WOG or OM samples (about 1 g or ml) were mixed with acidified methanol (methanol:water:HCl 80:19.9: 0.1 v/v), homogenized at 12,000 rpm for 2 min, and agitated for 16 h. Then, the supernatants of all samples were collected after centrifugation at 5000 rpm for 15 min at 4°C. These extracts were used to determine TPC and evaluate antioxidant activity.

### 2.7 Determination of total phenolic content (TPC)

The TPC was determined by Folin–Ciocalteu's reagent method according to a procedure described by Best et al. (2020). Briefly, 100 μl of extracts was mixed with 750 μl of Folin–Ciocalteau reagent (0.2 N). After incubation for 5 min at room temperature, 750 μl of sodium carbonate (7.5%) was added and incubated in a water bath at 40°C for 30 min. The OD was recorded at 725 nm, and the results were calculated in mg of gallic acid eq/kg or L of the sample by plotting the standard curve using different concentrations of gallic acid as standard.

### 2.8 Evaluation of antioxidant activity

The antioxidant activity (AOA) of the materials was evaluated using 1,1‐diphenyl‐2‐picrylhydrazyl (DPPH; Sigma Aldrich Corp., St. Louis, Mo, USA). Briefly, 50 μl of extracts were mixed with 950 μl of DPPH solution (65 μmol/L in methanol) and incubated at room temperature for 10 min. The OD of the samples (As) was measured at 515 nm by using a microplate reader. DPPH solution without the sample was used as blank (Ab) (Best et al., 2020; Rahmani et al., 2021). DPPH scavenging activity (%) was calculated by Equation (4):

(4) $$\text{DPPH scavenging activity}\;\left(\%\right)=\left(\left(\mathrm{Ab}-\mathrm{As}\right)/\mathrm{Ab}\right)\times 100$$

The butylated hydroxytoluene (BHT) was used as a standard antioxidant. The AOA of the samples was obtained by plotting the standard curve of DPPH scavenging activity (%) against the different concentrations of BHT and expressed as mg BHT eq/L or kg.

### 2.9 Sensory evaluation

Sensory attributes, including color, aroma, flavor, and overall acceptability of nine different OM, were performed by 30 semitrained panelists, including students and staff of the Ardabil University of Medical Sciences (aged between 19 and 40 years) by using a 9‐point hedonic scale (1 = dislike extremely, 2 = dislike very much, 3 = dislike moderately, 4 = dislike slightly, 5 = neither like nor dislike, 6 = like slightly, 7 = like moderately, 8 = like very much, and 9 = like extremely). After adjusting the temperature and the pH to 25°C and 6.7, respectively, the samples were served in clear plastic cups containing 25 ml of products with a random three‐digit code at room temperature under normal daylight conditions. Filtered water was provided for panelists to rinse their mouths to minimize residual effects. Informed consent was obtained from all panelists.

### 2.10 Statistical analysis

All analyses of OM treatments (*n* = 9) were performed in triplicate (*n* = 3), and results were expressed as means ± standard deviations. Data were statistically analyzed by one‐way analysis of variance (ANOVA) using SPSS for Windows (Version 22.0, SPSS Inc., Chicago, IL, USA). Duncan's test was applied to determine significant differences (*p* < .05). The sensory evaluation results were analyzed by the nonparametric Kruskal–Wallis test, followed by paired comparison using the Mann–Whitney U test.

## 3 RESULTS AND DISCUSSION

In this research, the effect of single and combination of acid, alkaline, α‐amylase, and sprouting treatments on the nutritional and functional properties of OM, including milk and protein extraction yields, protein, fat, carbohydrates, energy value, starch and reducing sugar, total phenolic content, and antioxidant activity, and also sensorial properties of OM were studied. The chemical composition of WOG is given in Table 1. The DM content of the WOG of our study was 94.56%. The high content of DM, along with low moisture content was considered the main factor for storage capability for cereal grains (Welch, 2011). In addition, carbohydrates, starch, and proteins of WOG were 61.55%, 39.25%, and 18.73%, respectively. Similarly, Russo et al. (2016) reported that DM, carbohydrate, and protein concentrations of milled WOG from Sweden were 93%, 61.5%, and 13.4%, respectively. Furthermore, Sterna et al. (2016) reported that the protein content of five different oat grains ranged from 9.7% to 17.30%. Moreover, the starch content of oat grains in their study ranged from 27.3% to 50.01%. These factors are reported as the main composition of oat grains and affect their functional properties (Peterson, 1992). Also, Demi̇r et al. (2021) reported that the carbohydrate and protein concentrations of oatmeal were 53% and 14%, respectively. The fat content and energy value (EV) of the WOG in our study were 6.12% and 376.22 Kcal/100 g, respectively. Bekers et al. (2001), Russo et al. (2016), and Demi̇r et al. (2021) reported that the fat content of oat grains was 6.2%, 7%, and 7.5%, respectively. Also, Russo et al. (2016) reported that the EV of WOG was 360 Kcal/100 g. It was reported that the fat content of oat grains is mostly higher than other cereals leading to their higher energy content (Welch, 2011). Moreover, the ash and reducing sugar content of WOG in our study were 8.16% and 5.17%. Our results were higher than those of Patel et al. (2008), who reported that the ash and reducing sugar content of spent oat were 1.86% and 1.37%, respectively. The lower ash and reducing sugar content in previous reports (Hitayezu et al., 2015; Sandhu et al., 2017) may be due to the dehulling of oat grains in those studies.

**TABLE 1:** The chemical composition of the whole‐oat grain

Component of WOG	
Protein (%)	18.73 ± 0.23
Fat (%)	6.12 ± 0.11
Carbohydrates (%)	61.55 ± 0.19
Dry matter (%)	94.56 ± 0.39
Ash (%)	8.16 ± 0.051
Starch (%)	39.25 ± 0.53
Energy (Kcal/100 g)	376.22 ± 1.85
Reducing sugar (%)	5.17 ± 0.05
Total phenolic content (mg GAE/kg)	1929.17 ± 41.25
Antioxidant activity (mg BHT eq/L)	700.10 ± 80.52

The TPC and antioxidant activity of WOG were 1929.17 mg GAE/kg and 700.1 mg BHT eq/kg, respectively. The results are in agreement with the result of Xu et al. (2009), who reported that the TPC of naked oat was 1650 μg/g. Furthermore, Sandhu et al. (2017) reported that the TPC of five different Indian oat cultivars ranged from 1744 to 2687 μg/g. In addition, the results show that the TPC value of WOG in this study was higher than the results of Emmons and Peterson (1999). They reported that the TPC and antioxidant activity of five different cultivars of oat grains from the United States ranged from 238 to 278 mg/kg and from 30.5% to 57.5%, respectively. Furthermore, the results of this study were higher than the results of Călinoiu et al. (2019), who reported that the TPC of oat grain was 211 mg/kg. Also, Sandhu et al. (2017) reported that oxidation inhibitory properties of oat grains ranged from 11.2% to 15.3%. Many researchers reported that the TPC and AOA of oat grains are affected by genotype, location, and storage time (Alfieri & Redaelli, 2015; Antonini et al., 2016; Menga et al., 2009). The differences in the composition of WOG in this study and previous reports may be related to the genetic varieties and environmental (Antonini et al., 2016; Sterna et al., 2016) and cultivation conditions (Lapveteläinen et al., 2001). It was reported that environmental factors were more effective than genetic varieties in the composition of oat grains (Hutchinson & Martin, 1955).

**FIGURE 1:**
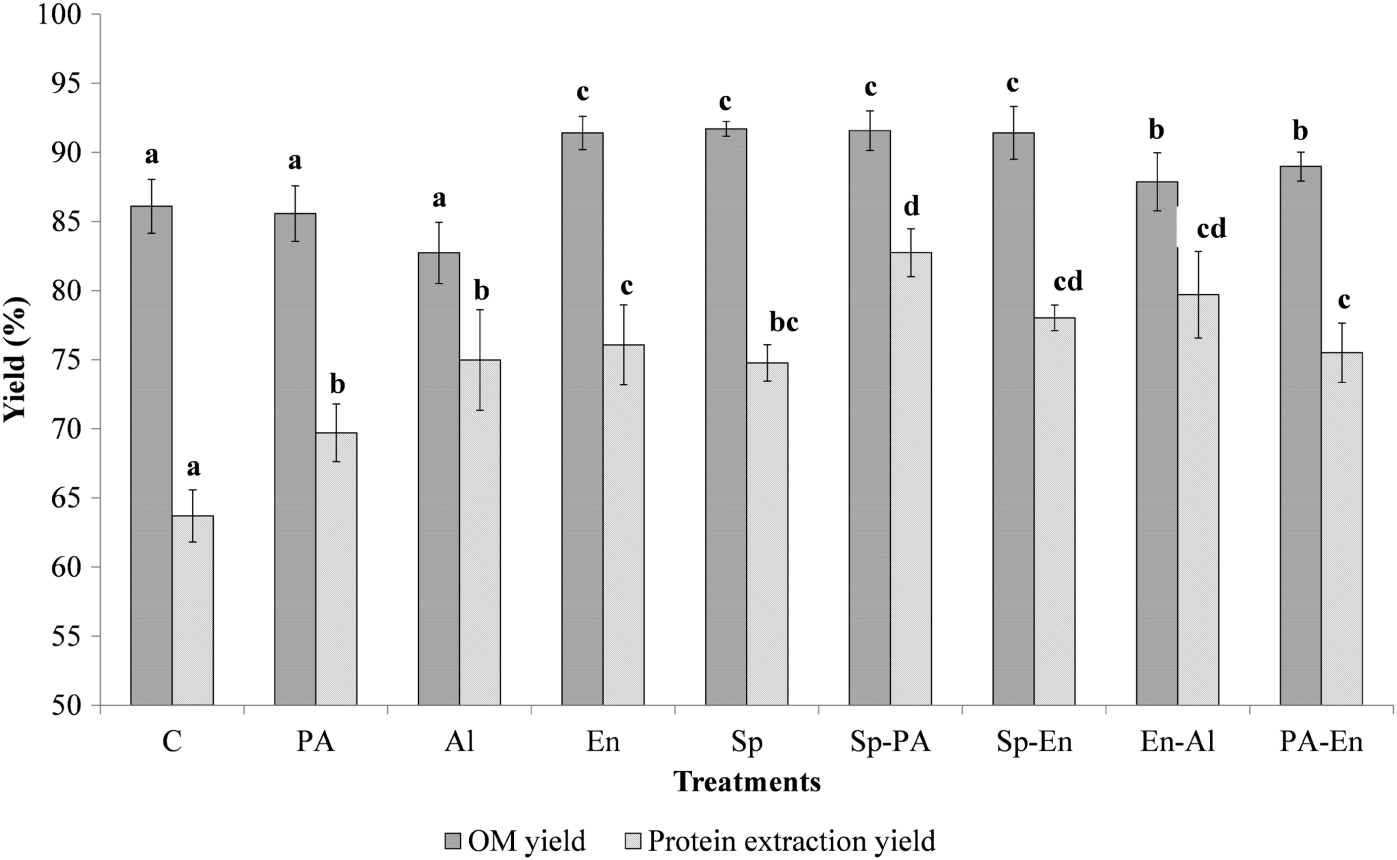
The oat milk (OM) yield and protein extraction yield of different treatments of oat milk (control (C), acid (PA), alkaline (Al), enzyme (En), sprouting (Sp), sprouting–acidic (Sp‐PA), sprouting–enzyme (Sp‐En), enzyme–alkaline (En‐Al), and acidic–enzyme (PA‐En)). For each parameter, different letters indicate significant differences at the 5% level.

The OM yield and PEY of different OM treatments are shown in Figure 1. The yield of OM produced by different treatments ranged from 82.73% (Al) to 91.70% (Sp). These results are in agreement with the results of Salama et al. (2011), who reported that the yields of different OM treatments ranged from 87.6% to 91.25%. However, this study's results were higher than those of Deswal et al. (2014), who reported extraction yield of OM produced by starch liquefaction with α‐amylase treatments ranged from 53.92% to 78.87%. Also, to our best knowledge, the effect of two‐stage treatments on the yield of OM had not been evaluated before. Results showed that the yield of Sp, En, and all two‐stage treatments was significantly higher than other treatments (*p* < .05). It was confirmed that the yield of OM was strongly affected by viscosity decreasing by starch digestion and prevention of its gelatinization during thermal processing and filtration during the treatments such as sprouting and α‐amylase digestion (Bekers et al., 2001; Tian et al., 2010). Moreover, the yield of OM could be increased by dietary fiber digestion during sprouting and reduction in water‐binding capacity properties (Hübner & Arendt, 2013; Tian et al., 2010).

The PEY of OM varied from 63.69% for control treatment to 82.7% for Sp‐PA treatments. Salama et al. (2011) reported a lower PEY value (43.15%–35.97%) despite higher OM yield, which may be due to the lower protein concentration of oat grains (10.72%) compared to the results of this study. The highest PEY value was achieved by most of the two‐stage treatments. Also, the low PEY value in PA treatment may be due to the aggregation and precipitation of proteins in acidic conditions (Kehinde et al., 2020; Loponen et al., 2007). Furthermore, in the case of Sp‐PA treatment, the high value of PEY was achieved despite the acidic condition. It has been reported that the increase in free amino nitrogen during sprouting may affect protein solubility (Tian et al., 2010). Moreover, the alkaline treatment had a low OM yield, despite the high protein content, which led to a lower PEY value.

The protein, fat, carbohydrates, DM, ash, and EV content of OM from different treatments are given in Table 2. The protein concentration of Al, Sp‐PA, and En‐Al treatments of OM was 1.72% which was significantly higher than other treatments (*p* < .05), while the control treatment showed the lowest protein concentration (1.41%). However, there is no study on the evaluation of the effect of two‐stage treatments on the production of OM; the protein concentration of all treatments showed higher value compared to the results of previous studies, which ranged from 0.63% to 1.32% (Bernat et al., 2015; Collard & McCormick, 2021; Mårtensson et al., 2000; Ramzan et al., 2021; Ravindran & RadhaiSri, 2021; Zhang et al., 2007). This difference in reports may be due to the differences in protein concentration of oat grains and/or differences in OM treatments and the ratio of oat to water (Salama et al., 2011). Results showed that the protein extraction was high in alkali conditions, which is in agreement with the results obtained by Guan and Yao (2008) and Wang et al. (1999). This phenomenon may be due to the cell wall disruption properties (Nascimento et al., 2016) and also changes in ionic or other surface properties of proteins (Wang et al., 1999). Furthermore, the results of this work show that two‐stage treatments of OM have a relatively high protein concentration. It could be due to the disruption of aleurone cell walls by different treatments and more heating steps (Verma et al., 2018).

**TABLE 2:** The chemical composition of OM produced by different treatments (mean ± *SD*)

Treatments	Protein (%)	Fat (%)	Carbohydrates (%)	DM (%)	Ash (%)	Energy value (Kcal/100 ml)
C^1^	1.41 ± 0.04^a^	0.21 ± 0.01^b^	4.20 ± 0.25^ab^	6.02 ± 0.31^ab^	0.20 ± 0.04^a^	24.32 ± 1.18^ab^
PA	1.55 ± 0.05^b^	0.45 ± 0.02^f^	4.21 ± 0.40^ab^	7.32 ± 0.17^d^	1.11 ± 0.21^e^	27.09 ± 1.31^bc^
Al	1.72 ± 0.01^d^	0.66 ± 0.04^i^	3.73 ± 0.27^ab^	6.69 ± 0.39^bcd^	0.58 ± 0.11^cd^	27.73 ± 1.32^c^
En	1.58 ± 0.05^bc^	0.25 ± 0.01^c^	3.74 ± 0.35^ab^	6.28 ± 0.47^abc^	0.70 ± 0.05^d^	23.54 ± 2.06^a^
Sp	1.55 ± 0.04^b^	0.28 ± 0.01^d^	3.65 ± 0.21^ab^	5.68 ± 0.14^a^	0.19 ± 0.05^a^	23.36 ± 0.68^a^
Sp‐PA	1.72 ± 0.04^d^	0.50 ± 0.03^g^	3.52 ± 0.38^a^	6.76 ± 0.45^cd^	1.01 ± 0.03^e^	25.48 ± 1.83^abc^
Sp‐En	1.62 ± 0.02^c^	0.11 ± 0.01^a^	4.11 ± 0.40^ab^	6.23 ± 0.44^abc^	0.38 ± 0.03^b^	23.92 ± 1.71^a^
En‐Al	1.72 ± 0.04^d^	0.35 ± 0.02^e^	4.35 ± 0.21^b^	6.95 ± 0.28^cd^	0.52 ± 0.09^bc^	27.46 ± 0.96^c^
PA‐En	1.61 ± 0.03^c^	0.54 ± 0.02^h^	4.15 ± 0.51^ab^	7.41 ± 0.46^d^	1.10 ± 0.05^e^	27.92 ± 2.28^c^

*Note*: Values in each column followed by the different letters are significant at the 5% level.

^1^ Treatments: Control (C), acid (PA), alkaline (Al), enzyme (En), sprouting (Sp), sprouting–acidic (Sp‐PA), sprouting–enzyme (Sp‐En), enzyme–alkaline (En‐Al), and acidic–enzyme (PA‐En).

Results of this study show that the fat and carbohydrate content of different OM varied from 0.11% to 0.66% and 3.52% to 4.35%, respectively. Furthermore, the DM and ash content of OM produced by different treatments ranged from 5.68% to 7.41% and 0.19% to 1.11%, respectively. In addition, the EV of OM from different treatments ranged from 23.36 kcal to 27.92 kcal/100 ml. Previous studies reported varying results for OM composition produced by different treatments. Bernat et al. (2015) reported that DM, fat, and ash content of OM from peeled oat (oat:water 8:100) were 6.5%, 0.094%, and 0.099%, respectively. Furthermore, Ravindran and RadhaiSri (2021) reported that the DM, fat, carbohydrates, and EV of nonfermented OM were 8.01%, 0.37%, 7.3%, and 32.9 kcal/100 ml, respectively. Also, Ramzan et al. (2021) reported that the DM, fat, and ash content of OM were 2.55%, 0.09%, and 0.32%, respectively. Results showed that the fat content of PA, Al, Sp‐PA, En‐Al, and PA‐En treatments was significantly higher than other treatments (*p* < .05). These results are in agreement with the result of Strange and Schaich (2007), who reported that hydrolyses led to high‐fat extraction due to the release of bound lipids with starches and proteins. Moreover, the fat content of the alkali treatment was significantly higher than other treatments (*p* < .05). Furthermore, higher‐fat content leads to higher EV, which is confirmed by the higher EV of those treatments compared to the other treatments. The DM and ash content of PA, Al, Sp‐PA, En‐Al, and PA‐En treatments were significantly higher than other treatments (*p* < .05).

**FIGURE 2:**
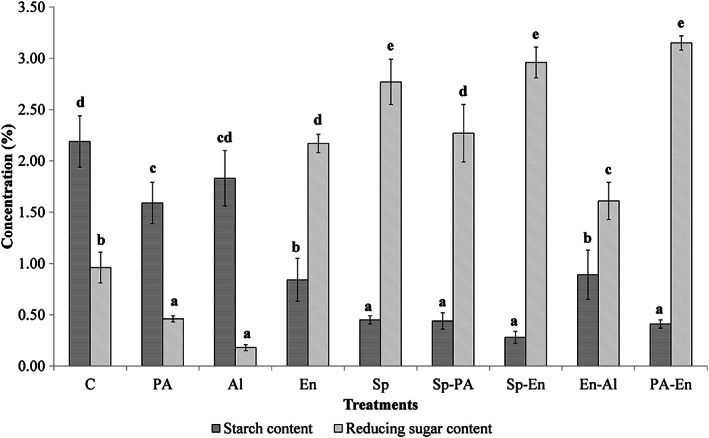
The starch and reducing sugar content of different treatments of oat milk (control (C), acid (PA), alkaline (Al), enzyme (En), sprouting (Sp), sprouting–acidic (Sp‐PA), sprouting–enzyme (Sp‐En), enzyme–alkaline (En‐Al), and acidic–enzyme (PA‐En)). For each parameter, different letters indicate significant differences at the 5% level.

The starch and reducing sugar content of different OM treatments are shown in Figure 2. The starch content of different OM treatments differed from 0.28% for the Sp‐En treatment to 2.19% for the control treatment. The oat grains starch was digested during all treatments. The starch digestion during diluted phosphoric, α‐amylase, or sprouting treatments was reported by previous studies (Aparicio‐García et al., 2021; Bekers et al., 2001; Herrera‐Ponce et al., 2014; Tian et al., 2010). In addition, results showed that the starch content of the sprouting treatment was almost half of the enzyme treatments, which could be related to the longer sprouting time compared to the enzyme treatment. In addition, the Sp‐En treatment showed the lowest starch content compared to the other treatments due to the more effective digestion stages. The two‐stage treatment of WOG with phosphoric acid and α‐amylase leads to a significantly high reducing sugar value of 3.15%, which is in line with the results of Bekers et al. (2001), who reported that limited enzymatic hydrolyses of oat starch increased the reducing sugar value in the liquid fraction from 3.23% to 6.30%. Also, these results are in agreement with the results of Bernat et al. (2015) and Collard and McCormick (2021), who reported that the reducing sugar of OM was 0.047% and 3.08%, respectively. The differences in reducing sugar content of OM and also between the treatments depend on the concentration and type of carbohydrates of oat grains (Peterson, 1992) and also treatments and digestion of different components of oat grains such as starch and lignocellulosic fibers (Bekers et al., 2001; Dziekońska‐Kubczak et al., 2019; Nair et al., 2018).

**FIGURE 3:**
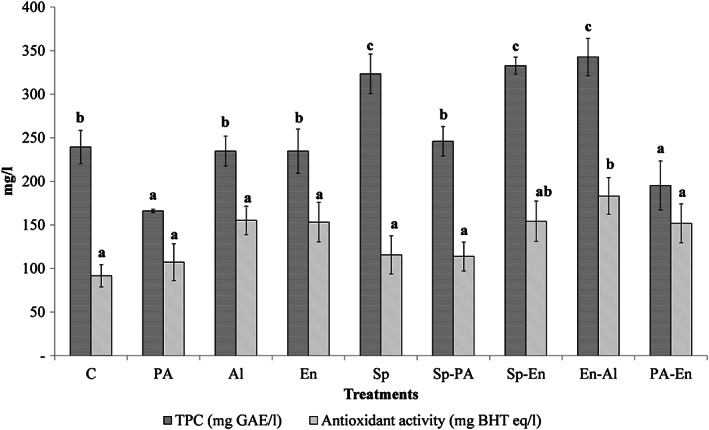
The total phenolic content (TPC, mg GAE/L) and antioxidant activity (AOA, mg BHT eq/L) of different treatments of oat milk (control (C), acid (PA), alkaline (Al), enzyme (En), sprouting (Sp), sprouting–acidic (Sp‐PA), sprouting–enzyme (Sp‐En), enzyme–alkaline (En‐Al), and acidic–enzyme (PA‐En)). For each parameter, different letters indicate significant differences at the 5% level.

The TPC and AOA of different OM treatments are shown in Figure 3. The TPC of OM differed from 166 mg GAE/L for PA treatment to 342.67 mg GAE/L for En‐Al treatments. Results showed that the TPC of OM was significantly affected by sprouting alone and combined with enzymatic and enzymatic–alkali treatments (*p* < .05). These results are in agreement with the results of previous studies, which reported that the TPC and antioxidant activity of oat were enhanced at least twofold by sprouting (Aparicio‐García et al., 2021; Rico et al., 2020), α‐amylase (Bei et al., 2018), and also acid and alkali treatments (Kim et al., 2006). They reported that alkali treatments have a higher value of TPC, which is in line with our results. The results of the present study showed a decreasing trend of phenolic acids after PA treatments which is reported by previous studies (Chen et al., 2016; Xu et al., 2009). Furthermore, the degradation of some phenolic compounds under hot acidic conditions was confirmed by previous studies (Kim et al., 2006; Verma et al., 2009). The AOA of different OM differed from 91.57 mg BHT eq/L for control treatment to 183.08 mg BHT eq/L for En‐Al of OM treatments. Xu et al. (2009) reported that the AOA of oat was enhanced during steeping and germination. It was reported that the liberation of phenolic compounds with the ability of hydrogen atoms or electron donation of hydroxyl groups of those for neutralizing free radicals could be responsible for the enhancement of the antioxidant activity of OM (Ding et al., 2019; Xu et al., 2020). The increase in AOA during germination may be not only related to the release of bounded phenolic compounds but also related to the de novo synthesis of avenanthramides (Ding et al., 2019). In addition, Aparicio‐García et al. (2021) reported a threefold increase in the AOA of oat grains after germination. It is worth noting that heating treatments such as roasting (Sandhu et al., 2017) by cell wall disruption (Chen et al., 2016) affect the extraction yield of TPC with a higher AOA value of samples. Also, the higher AOA value of En‐Al treatment may be related to the higher value of TPC. Nevertheless, results showed no relation between TPC and AOA changing OM. Results showed that on the contrary, decreasing the TPC value of PA treatment by around 30%, the AOA value was enhanced by 17% compared to the control treatment. Moreover, the same phenomenon happened in the PA‐En treatment. There are many reasons which make an ambiguous relation between the TPC and AOA of cereal grains, such as nonphenolic compounds with antioxidants activity (e.g., ascorbic acids, tocopherol, β‐glucan oligosaccharides, etc.), the interaction of antioxidants, and different assay methods (Sun et al., 2020), and also the different antioxidant activity of phenolic compounds like ferulic acids which showed low antioxidant properties (Brand‐Williams et al., 1995).

**FIGURE 4:**
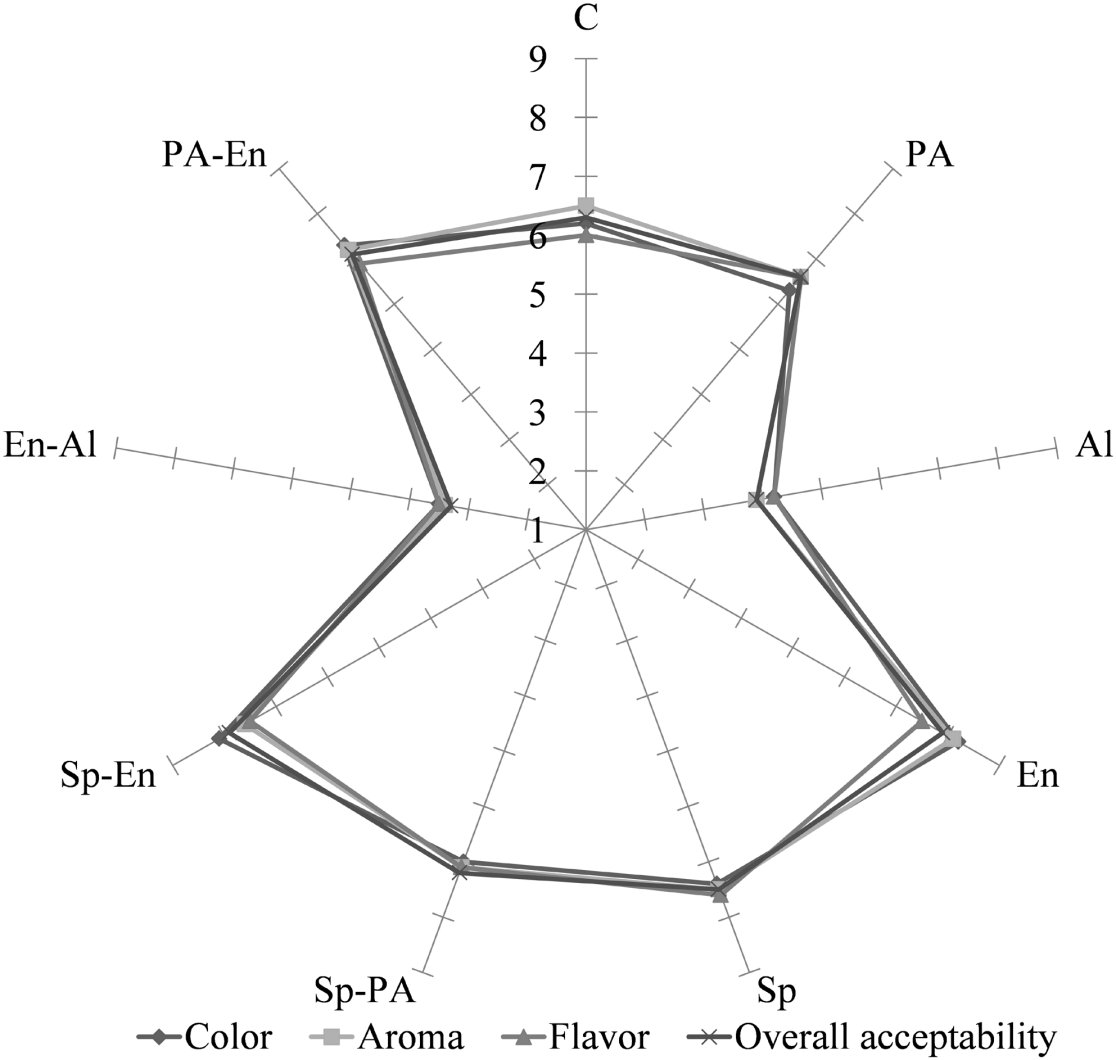
The sensorial properties (color, aroma, flavor, and overall acceptability) of different treatments of oat milk (control (C), acid (PA), alkaline (Al), enzyme (En), sprouting (Sp), sprouting–acidic (Sp‐PA), sprouting–enzyme (Sp‐En), enzyme–alkaline (En‐Al), and acidic–enzyme (PA‐En)).

The results of the sensorial properties of different OM treatments are shown in Figure 4. Results show that different treatments of OM significantly affect all OM sensory properties (*p* < .05). According to the sensory evaluation results, not only did the treatments of the enzyme, sprouting, Sp‐PA, Sp‐En, and PA‐En have significantly higher sensory scores compared to the other OM treatments but also the results showed that all sensory scores of these treatments were higher than 7 (like moderately), which is the important score for consumer acceptability and marketability point of view (Mridula & Sharma, 2015). Just in the case of the PA‐En treatment, the flavor score was 6.9, which did not show a significant difference between the mentioned treatments (*p* ≥ .05). The En treatment showed the highest color and aroma scores (8.2 and 8.1, respectively), and the sprouting treatment showed the highest flavor score (7.6). Furthermore, the overall acceptability score of enzyme and sprouting–enzyme treatments was the highest (7.9). The results showed that all sensory attributes of Al and En‐Al treatments were less than 4.2 (dislike slightly), which is not favorable for the marketability of those. During alkali extraction, the color of the slurry was changed to dark, which may be related to the production of protein–polyphenol interactions or oxidation of phenolic compounds (Wanasundara et al., 2017). Moreover, it is reported that this color change may be due to the color changing of pH depending on the anthocyanins of oat (Luana et al., 2014).

## 4 CONCLUSION

The results of this study revealed that using different treatments for the production of OM had different effects on the nutritional properties of OM, such as yield, protein concentration, and its yield, DM, fat, carbohydrate, starch, reducing sugar, energy content, as well as functional properties including phenolic compounds and antioxidant properties of OM. From the nutritional and technical point of view, sprouting and α‐amylase have the most effect on the protein, TPC, antioxidant properties, and the yield PEY of OM. Furthermore, dilute acid or alkali treatments combined with other treatments were more effective than singular treatments on investigated factors. Generally, treatments with high oat compound digestion, especially starch, could result in higher nutritional and functional properties of OM.

## CONFLICT OF INTEREST

The authors confirm that they have no conflicts of interest with respect to the work described in this manuscript.

## ETHICAL APPROVAL

All panelists enrolled in this study provided written informed consent. Also, they were informed that they can withdraw from the evaluation at any time without giving a reason.

## Data Availability

There are no primary data associated with this manuscript.
